# Chemical Synthesis of High-Stable Amorphous FeCo Nanoalloys with Good Magnetic Properties

**DOI:** 10.3390/nano8030154

**Published:** 2018-03-09

**Authors:** Bai Yang, Yue Wu, Xiaopan Li, Ronghai Yu

**Affiliations:** Key Laboratory of Aerospace Advanced Materials and Performance, Ministry of Education, School of Materials Science and Engineering, Beihang University, Beijing 100191, China; xxpbcxhs@buaa.edu.cn (Y.W.); xiaopan-lee@buaa.edu.cn (X.L.); rhyu@buaa.edu.cn (R.Y.)

**Keywords:** amorphous FeCo nanoalloys, chemical reduction, good air stability, high thermal stability, good magnetic properties

## Abstract

It is difficult to fabricate high-purity amorphous FeCo alloys by traditional physical methods due to their weak glass forming ability. In this work, the fully amorphous FeCo nanoalloys with high purity and good stability have been prepared by a direct chemical reduction of Fe^2+^ and Co^2+^ ions with NaBH_4_ as the reducing agent and polyvinylpyrrolidone (PVP) as the surfactant. The morphologies, surface compositions and particle sizes with their distribution of these amorphous samples can be effectively tuned by the suitable PVP additions. High crystallization temperature up to 468 °C, high saturation magnetization of 196.2 A·m^2^·kg^−1^ and low coercivity of 83.3 Oe are obtained in amorphous FeCo nanoalloys due to their uniform distribution, weak surface oxidation and low surface B concentration. Good frequency-dependent magnetic properties can be also achieved in the fully compacted amorphous sample with a high density of 7.20 g/cm^3^. The simple chemical method, high stability and good magnetic properties for these amorphous FeCo nanoalloys promise their significant potential applications in high-power magnetic devices.

## 1. Introduction

The nanosized FeCo or FeCo-based alloys have attracted much attention on their fabrication and characterization due to their good chemical properties and tunable magnetic properties varying with their compositions, which show a significant potential application in many technological areas such as numerous advanced magnetic devices, catalytic and medical applications [1,2,3,4]. In particular, compared with FeCo-based crystalline alloys, the amorphous counterparts have attracted more research interests for their combined good mechanical, electrical and magnetic properties including good deformability, high fracture strength, high resistivity and low coercivity, which make them a crucial soft magnetic material for high-power and high-frequency applications [5,6,7,8]. Commonly, these amorphous alloys based on FeCo can be fabricated by some traditional physical methods such as copper mold casting, rapid quench and single-roller melt spinning [9,10,11]. To improve the glass forming ability and increase the critical sizes of bulk FeCo-based amorphous alloys, some amorphous-promoting elements such as Si and B should be added in pure metal alloys [12,13,14,15]. The chemical method has also been utilized successfully to fabricate the amorphous metal-boron (TM-B, TM = Co, Ni, Fe) powders through chemical reduction of metal ions with KBH_4_ in aqueous solution [16]. It has been reported that Fe- or Co-based amorphous alloys can be also fabricated through arc melting by the addition of some rare earth elements such as Tb and Nd [17,18]. Without addition of these glass-formation elements, it is very difficult to fabricate metal-based amorphous alloys due to their poor glass forming ability. However, the additions of these non-magnetic or easily-oxidized glass-formation elements always introduce contamination and structural instability to the FeCo-based alloys, which would weaken their magnetic performances and the other physical or chemical properties [19].

So far, the fabrication of pure amorphous FeCo alloys has been seldom reported because of their weak glass forming ability. In our previous work, we have prepared high-purity amorphous Fe nanoparticles by a chemical method [20]. Our recent work have also revealed that the near-spherical micron-sized FeCo particles with high purity can be fabricated by a direct chemical reduction of Fe^2+^ and Co^2+^ ions with hydrazine hydrate as the reductant in a microemulsion system [21]. Consequently, there is still a challenge to explore an effective route for fabricating pure amorphous FeCo alloys with good stability and enhanced magnetic properties.

As is known, the magnetic and mechanical properties of FeCo alloys are greatly affected by their Fe/Co ratios and microstructure [22]. Our previous study has shown that high magnetization saturation (215 A·m^2^·kg^−1^) and low coercivity (73.4 Oe) can be obtained in the chemically synthesized Fe_65_Co_35_ micro-sized particles [23]. In this work, we have reported a facile chemical method to fabricate high-purity amorphous Fe_65_Co_35_ nanoalloys with slight self-surface modification. The microstructures along with their thermal stability, surface element states and magnetic properties of these amorphous FeCo nanoalloys have been systematically studied. 

## 2. Experimental

### 2.1. Synthesis of Amorphous FeCo Nanoalloys

**Figure 1:**
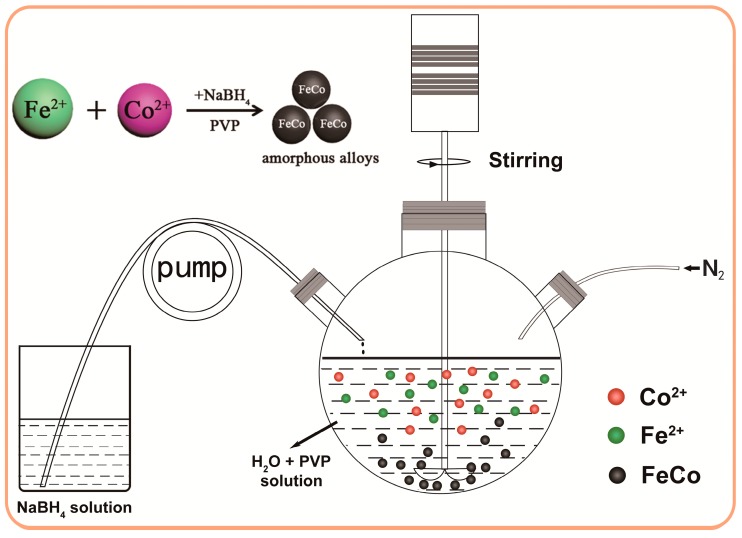
The schematic formation process for the amorphous FeCo nanoalloys.

The fully amorphous FeCo nanoparticles based on the nominal composition of Fe_65_Co_35_ with high purity and controlled morphologies have been fabricated by a directed reduction reaction of Fe^2+^ and Co^2+^ ions with the additions of NaBH_4_ as a reducing agent and PVP as surfactant, the schematic formation process for which is shown in Figure 1. A typical procedure is described as follows. Firstly, the suitable amount of FeCl_2_·4H_2_O (1.2923 g), CoCl_2_·6H_2_O (0.8323 g) and PVP (1.5 g) were dissolved in distilled water (50 mL) in a three-necked round-bottom flask. Meanwhile, the NaBH_4_ (0.7562 g) was dissolved in distilled water (40 mL) in another flask. Then, high-purity N_2_ gas was continuously bubbled into the mixed solution containing Co^2+^ and Fe^2+^ ions for 10 min to remove the dissolved oxygen in the mixed liquid. Thirdly, the solution containing NaBH_4_ was added dropwise into the three-necked round-bottom flask containing Co^2+^ and Fe^2+^ ions at a rate of 4 mL/min, through a constant-flow pump when stirring vigorously. The reaction process would persist for 50 min and should sustain under high-purity N_2_ atmosphere up to the completion of the reaction. The reaction process can be summarized with the following equation:

Fe^2+^ + Co^2+^ + 4 BH_4_^−^ + 12 H_2_O → FeCo + 4 B(OH)_3_ + 14 H_2_↑ (1)

Finally, the black precipitates were collected by an external magnet after washing them for several times with distilled water and ethanol in sequence and then dried in a vacuum oven at 25 °C for 12 h to get the final products. For further investigation of the effects of different PVP additions on the microstructures for these amorphous FeCo alloys, the other amorphous samples based on the same nominal composition of Fe_65_Co_35_ but fabricated without and with PVP additions of 1 and 2 g were also prepared. To investigate crystallization characteristics of these amorphous samples, the as-synthesized sample prepared with 1.5 g PVP additions was annealed under high-purity Ar atmosphere at different temperature from 300 to 550 °C for 10 min. 

### 2.2. Materials Characterization

The phase structures of the samples were characterized by X-ray diffraction (XRD, Rigaku Corporation, Tokyo, Japan) using a D/max 2500PC X-ray diffractometer (Cu Kα radiation, λ = 1.5406 Å). The microstructures of the samples were carried out by a JEOL JEM-2100 transmission electron microscope (TEM, JEOL Co., Ltd., Tokyo, Japan) operated at 200 kV along with a selected-area electron diffraction (SAED). An energy dispersive spectroscope (EDS, JEOL Co., Ltd., Tokyo, Japan) was also used to determine the chemical elements in the products by the same TEM. An inductively coupled plasma optical emission spectroscopy (ICP-OES, Optima 7000 DV, Perkin-Elmer, Waltham, MA, USA) was used to detect the precise compositions of the samples. The thermal properties of these amorphous alloys were analyzed by a differential scanning calorimetry (DSC, STA 449 F3, NETZSCH-Gerätebau GmbH, Selb, Germany) under an Ar atmosphere at a heating rate of 10 °C/min. The X-ray photoelectron spectroscopy (XPS, ESCALAB 250Xi, Thermo Fisher Scientific, Waltham, MA, USA) measurement was performed to detect the element states in the samples. The intrinsic magnetic properties of the samples were measured by a vibrating sample magnetometer (VSM, BHV-55, Riken Keiki Co., Ltd., Tokyo, Japan) with a maximum magnetic field of 10 kOe at room temperature. For measuring soft magnetic properties of the products, full-density toroidal samples with an outer diameter of 15 mm, an inner diameter of 9 mm and a sample thickness of 3 mm were produced by directly compacting the products under a static pressure of 1800 MPa. The density of the compacted samples was measured by the Archimedes method. The frequency-dependent magnetic properties for the toroidal samples were measured by an Agilent 4294A precision impedance analyzer (Agilent Technologies, Santa Clara, CA, USA) with a maximum operating frequency of 5 MHz.

## 3. Results and Discussion

### 3.1. Structural Analysis

**Figure 2:**
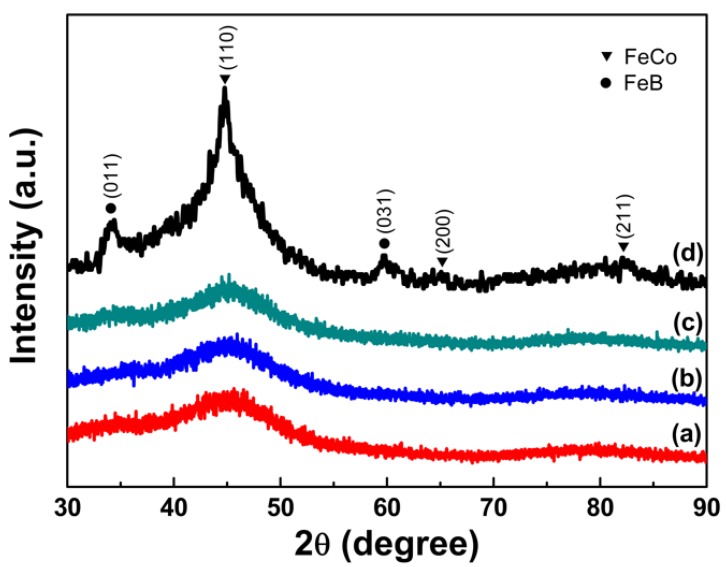
The X-ray diffraction (XRD) patterns of as-synthesized samples prepared with different PVP additions of 1 g (a), 1.5 g (b), 2 g (c) and without PVP (d).

Figure 2 shows the XRD patterns of as-synthesized samples prepared with PVP additions of different amount of 1 g (a), 1.5 g (b), 2 g (c) and without PVP (d) addition. It can be seen that all the three samples prepared with PVP additions exhibit almost the same diffraction characteristics with a broad peak appearing at 2θ of approximately 45° indicating the formation of a fully amorphous phase [24]. The amorphous state of the samples can be further confirmed by their SAED patterns and crystallization characteristics in DSC curves discussed later. However, from the XRD patterns for the sample prepared without PVP addition shown in Figure 2d, four broad peaks can be observed with 2θ values of approximately 34.2°, 44.7°, 59.7° and 82.4°, which can be indexed to FeB and FeCo, respectively. It can be concluded that the PVP additions as the surfactant in the chemical reduction can facilitate the formation of amorphous FeCo alloys. The PVP additions may probably suspend the co-reduction of Fe^2+^ and Co^2+^ ions and restrain their alloying to crystalline FeCo phase so as to promote the formation of amorphous FeCo phase.

**Figure 3:**
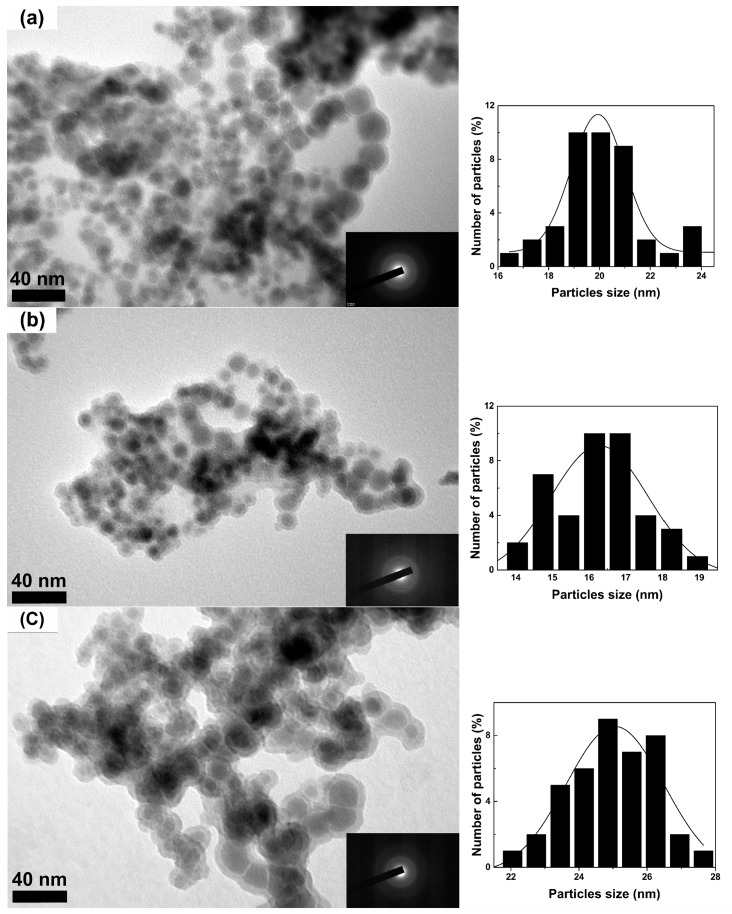
The representative transmission electron microscope (TEM) micrographs and the selected-area electron diffraction (SAED) patterns along with the corresponding histograms on the right side of the figure for amorphous FeCo nanoparticles prepared with different PVP additions of 1 g (**a**); 1.5 g (**b**) and 2 g (**c**).

Figure 3 shows the representative TEM micrographs and the SAED patterns along with corresponding histograms for the three amorphous samples prepared with different PVP additions. The number of particles used for the histograms in Figure 3 is approximately 300 for every sample. As shown in Figure 3, the SAED patterns for the three samples only show the diffuse scattering halos indicating the fully amorphous characteristics in these samples. Furthermore, these amorphous FeCo nanoparticles exhibit a nearly spherical shape with good dispersion. It can be seen the average size can be decreased from 20 to 16 nm for the samples prepared with increasing the PVP additions from 1 to 1.5 g. However, the sample prepared with much more PVP additions of 2 g exhibits much larger average size of 25 nm. The more monodisperse sample with narrow size distribution can be prepared with 1.5 g PVP additions. The actual Fe/Co/O ratios detected by EDS for these amorphous samples are listed in Table 1. Although the small amount of O is detected by EDS measurements, no oxidation can be found in XRD patterns, which indicates that very low surface oxidation occurs in these amorphous nanoparticles. It should be noted that the surface oxidation leads to the shell structure observed in the TEM images for these amorphous FeCo samples, which can be ascribed to the co-effect of the self-surface oxidation and the further oxidation from the preparation process for the TEM samples. It has been reported that the surface oxidation would inevitably occur in high-purity metal-based nanoparticles with small particle size of less than 30 nm and can prevent them from further oxidation in a short period [25]. The surface oxidation is often observed by the TEM but not by the XRD analysis due to their low content within the resolution limit of 5 at. % for the XRD measurement [26,27]. As is known, the TEM observed samples have been often prepared by dispersing the nanoparticles in a pure alcohol or acetone solution, then dropping the dispersed particles on a suitable copper net and finally drying them for TEM observation. Therefore, the preparation process for TEM samples probably increases the surface oxidation for the ultrafine metal nanoparticles due to their easy oxidation. Moreover, the surface oxidation occurring in the nanoparticles can act as the thin area for TEM observation and shows a higher contrast resolution than that for the unoxidized part due to the different structure of the two parts so as to exhibit a seeming shell on the nanoparticles. So a core-shell structure is often observed in TEM images for the ultrafine metal nanoparticles prepared in air. The similar oxide shells can be also observed in the TEM images but not detected by XRD measurement for the ultrafine high-purity Fe [26,28] or CoFe [29] nanoparticles with surface oxidation. The slight self-surface oxidation for amorphous FeCo nanoalloys can be ascribed to the unavoidable surface effects resulting from their small particle sizes and can be also detected by their XPS study discussed below.

### 3.2. Crystallization Characteristics

**Figure 4:**
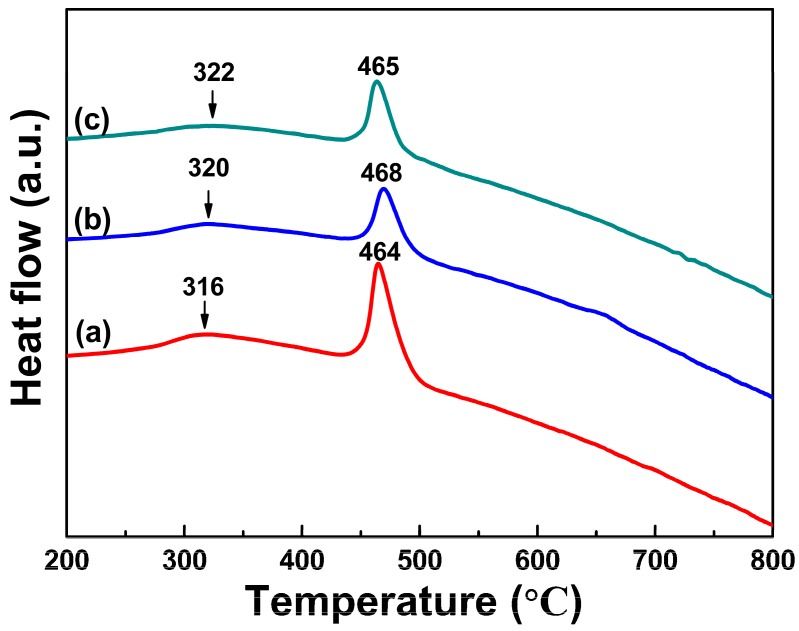
The DSC measurement of amorphous FeCo nanoparticles prepared with different PVP additions of 1 g (a), 1.5 g (b) and 2 g (c).

The DSC measurement was carried out to characterize the crystallization characteristics of the three amorphous samples prepared with different PVP additions. As shown in Figure 4, all the three samples exhibit similar thermal behaviors with a distinct exothermic peak at temperature of approximately 465 °C and another bump at temperature ranging from 316 to 322 °C in the DSC curves, which can be closely related to the two different crystallization process. As is known, the crystallization characteristics of amorphous materials are closely related with their compositions and amorphous phases. From Figure 2 and Table 1, the three amorphous samples exhibit nearly the same amorphous states and also have very similar compositions, which leads to their similar crystallization characteristics. From Figure 4, the obvious exothermic peak at high temperature is probably associated with the transformation from amorphous FeCo phase to the crystallized FeCo phase, and the exothermic peak at low temperature probably corresponds to another primary crystallization process from a fully amorphous phase to a primary amorphous phase as discussed below. The same crystallization characteristics can be also observed in the other FeCo-based amorphous alloys fabricated by traditional melt-spinning technique [30]. Compared with FeCo-based amorphous alloys with additions of amorphous-promoting elements such as B and Si, the amorphous FeCo nanoparticles exhibit relatively lower transition temperature due to their high-purity [31]. Among three amorphous samples, the sample prepared with 1.5 g PVP additions exhibits the highest transition temperature of 468 °C, which can be ascribed to their uniform particle sizes with narrow size distribution. Another bump-like exothermic peak at low temperature may correspond to the crystallization of primary amorphous phase discussed below.

**Figure 5:**
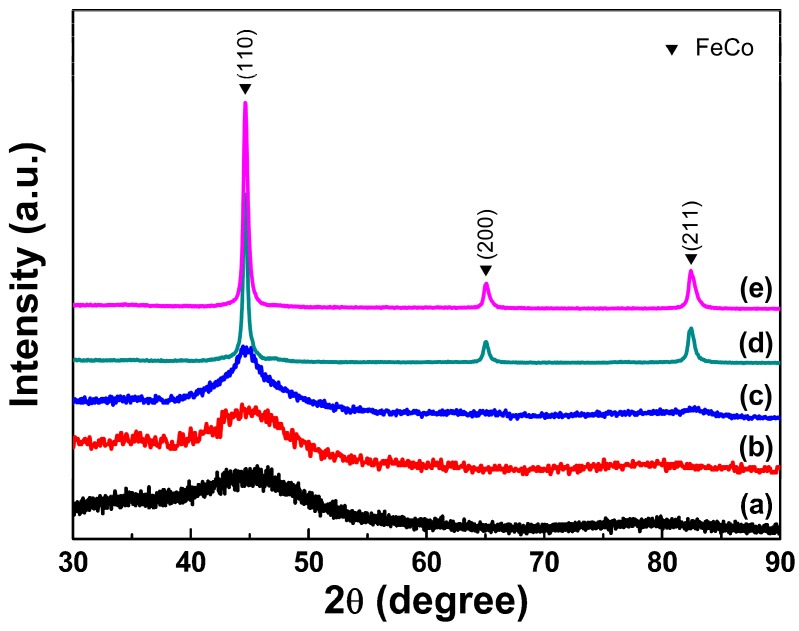
The XRD patterns of the original amorphous FeCo nanoparticles (a) prepared with 1.5 g PVP additions and the treated samples being annealed under Ar gas atmosphere at different temperature of 300 °C (b), 350 °C (c), 450 °C (d) and 550 °C (e) for 10 min.

To further investigate the detailed thermal transition characteristics for these amorphous FeCo nanoalloys, the typical sample prepared with 1.5 g PVP additions has been annealed under Ar gas atmosphere around the exothermic peaks in the DSC curves of 300, 350, 450 and 550 °C for 10 min. Figure 5 shows the XRD patterns of this sample annealed at different temperatures. As shown in Figure 5b,c, the treated samples annealed around low bump-like exothermic peak of 300 and 350 °C still remain fully amorphous states, which confirm the existence of primary amorphous phase in the original amorphous sample. As shown in Figure 5d,e, for the annealed samples treated around high exothermic peak of 450 and 550 °C, three diffraction peaks at 2θ values of 44.8°, 65.1°, and 82.4° corresponding to the crystal planes of (110), (200) and (211) can be observed in their XRD patterns, which are in good agreement with those of bcc-FeCo phase. It has been proved that the crystallized Co nanoparticles with different morphologies often exhibit stable fcc- or hcp-structure [32,33] and their XRD patterns are very different with those for bcc-FeCo phase. Otherwise, the bcc-Co nanoparticles can be obtained as a metastable structure with a strict size limitation of 2–5 nm [34]. Compared with XRD patterns for the original samples and the annealed ones, it can be found that not the other phases containing Co but the single bcc-FeCo phase in fully-annealed amorphous samples can be detected, which confirms the formation of high-purity amorphous FeCo phase but not a mixture of amorphous Co and Fe nanoparticles or amorphous Co@Fe core-shell nanoparticles in the as-synthesized samples prepared with PVP additions. Moreover, the fully-annealed sample treated at a high temperature of 550 °C exhibits a very similar XRD pattern (Figure 5d) with that of the annealed sample treated at 450 °C (Figure 5e) and only a pure bcc-FeCo phase can be observed in these two patterns, which further confirms the existence of high-purity amorphous FeCo in three as-synthesized amorphous samples. From Figure 5d, the actual transition temperature of the amorphous sample can be also detected to be around 450 °C, which accords well with the results of the above DSC measurement.

### 3.3. X-ray Photoelectron Spectroscopy (XPS) Surface Analysis

**Figure 6:**
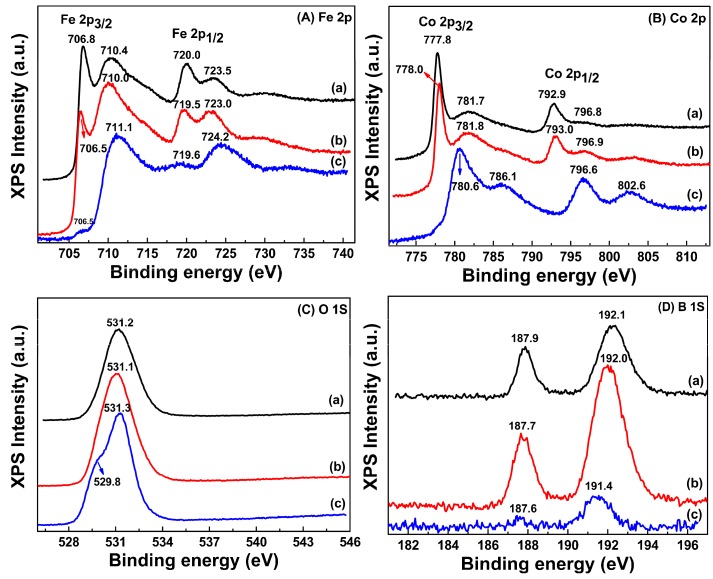
The XPS narrow-scan spectra of Fe 2p (**A**), Co 2p (**B**), O 1s (**C**) and B 1s (**D**) for amorphous FeCo nanoalloys prepared with different PVP additions of 1.5 g (a), 2 g (b) and the sample prepared without PVP (c).

The surface element states of Fe, Co, B and O atoms in the as-synthesized amorphous FeCo nanoparticles and another sample prepared without PVP addition have been detected by the XPS narrow-scan spectra shown in Figure 6. The similar surface element states can be observed in the amorphous samples prepared with different PVP addition of 1.5 and 2 g. It can be clearly found from Figure 6A that two Fe 2p peaks with Fe 2p_3/2_ at approximately 706.5 eV and Fe 2p_1/2_ at approximately 719.5 eV are observed for two amorphous samples indicating a pure metallic state of Fe atoms in them [35]. Moreover, the appearance of another two Fe 2p peaks with Fe 2p_3/2_ at approximately 710.0 eV and Fe 2p_1/2_ at approximately 723.3 eV demonstrates the existence of Fe_3_O_4_ phase in the two amorphous samples [36]. It can be found from Figure 6B that both of the two Co 2p spectra with an appearance of the Co 2p_3/2_ at approximately 778.1 eV and Co 2p_1/2_ at approximately 793.2 eV show a pure metallic Co state in the two amorphous samples [37]. Another two Co 2p peaks with Co 2p_3/2_ at approximately 782.1 eV and Co 2p_1/2_ at approximately 797.1 eV can be observed in Figure 6B indicating the presence of Co_3_O_4_ and can be further confirmed by their O 1s spectra with the peak around 530.7 eV shown in Figure 6C [38]. As shown in Figure 6D, the B 1s spectra for two amorphous samples also exhibit very similar curves and peak locations, which indicate the same B element state. The B 1s peaks at 192.1 and 187.9 eV can be attributed to the existence of FeB or CoB phases on surface of the amorphous samples [39,40]. The slight shift of peak location of 0.6 eV in XPS narrow-scan spectra for the two amorphous samples can be ascribed to of their different surface compositions, which are verified by the EDS investigation in Table 1. However, the Fe 2p spectra for the sample without PVP addition exhibit two Fe 2p peaks with Fe 2p_3/2_ at approximately 710.8 eV and Fe 2p_1/2_ at approximately 724.2 eV indicating the existence of Fe_3_O_4_ phase and while the appearance of two Co 2p peaks with Co 2p_3/2_ at 780.6 eV and Co 2p_1/2_ at 796.6 eV along with a broad bump at approximately 786.1 eV, which accords with those of CoFe_2_O_4_ phase [41]. Thus, no pure metallic Co but very weak pure metallic Fe state detected for the sample prepared without PVP addition indicates their more serious surface oxidation. It can be found that the PVP additions during the preparation can not only promote the formation of amorphous FeCo nanoparticles, but also weaken their surface oxidation.

As is known, the XPS investigation provides the surface element information of the samples for the photoelectron probing depth is within only a few angstroms. As discussed above, weak O and B concentration for amorphous FeCo nanoparticles is observed by XPS measurement. However, no oxidation or B-contained phases are detected by the XRD measurement for the original amorphous samples and the fully annealed ones. To make sure the precise compositions for these amorphous particles, the ICP-OES has been adopted to detect the elements in the samples. As shown in Table 1, besides Fe and Co elements, another B element is detected by ICP-OES, which however cannot be detected by EDS due to the low surface B concentration outside the measuring accuracy of EDS. Combined with the XPS, ICP-OES and XRD results, it can be deduced reasonably that self-surface modification including weak surface oxidation and low surface B concentration occurs in these chemically-synthesized amorphous FeCo nanoparticles. It has been reported that air stability for the chemically-synthesized Co nanoparticles with size range of 3–11 nm can be greatly improved by their gentle surface oxidation [42]. In this work, the as-synthesized products have been preserved without protective atmosphere after completion of their preparation and measured within a week. So the weak surface oxidation occurring in these amorphous FeCo nanoparticles can probably delay their further oxidation in air within a short period, for no oxidation can be detected by their XRD measurements. Furthermore, the low surface B aggregation can also protect these amorphous FeCo nanoparticles from further oxidation and lead to their good air and thermal stability [20]. From the above discussion, it can be concluded that the suitable PVP additions promote the formation of high-purity amorphous FeCo nanoalloys with slight self-surface modification. Furthermore, the different PVP amounts show an obvious influences on the particle sizes with their distribution and the Fe/Co ratios for these amorphous samples, and also protect the amorphous FeCo nanoalloys from further oxidation.

### 3.4. Magnetic Properties

**Figure 7:**
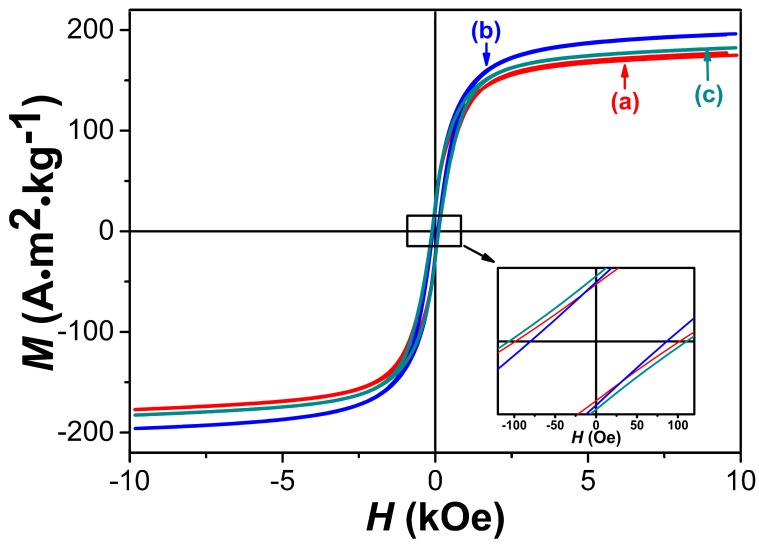
Magnetic hysteresis loops of amorphous FeCo nanoparticles prepared with different PVP additions of 1 g (a), 1.5 g (b) and 2 g (c).

Figure 7 shows the room-temperature hysteresis loops for the three amorphous FeCo samples. It can be found that all the three samples exhibit typical ferromagnetism with good intrinsic magnetic properties. The values of saturation magnetization *M*_s_ and coercivity *_i_H*_c_ of the three samples are also listed in Table 1. The high *M*_s_ values of 175.0, 196.2 and 182.3 A·m^2^·kg^−1^ are obtained for the samples prepared with PVP additions of 1, 1.5 and 2 g, respectively. Compared with the *M*_s_ value (215 A·m^2^·kg^−1^) of crystalline Fe_65_Co_35_ micro-sized particles, the slight decreasing of *M*_s_ for the amorphous FeCo nanoparticles can be ascribed to their surface oxidation along with surface B concentration and the spin canting effect for nanoparticles [43,44]. The product prepared with 1.5 g PVP additions exhibits highest *M*_s_ value of 196.2 A·m^2^·kg^−1^ among the three samples due to the low surface O and B content as shown in Table 1. It can be also found from Table 1 that these amorphous samples exhibit relatively high coercivity of 83.3–108.5 Oe by virtue of their smaller particle sizes and surface oxidation. The amorphous FeCo nanoparticles prepared with 1.5 g PVP addition also exhibit lowest *_i_H*_c_ value of 83.3 Oe due to their narrow size distribution.

**Figure 8:**
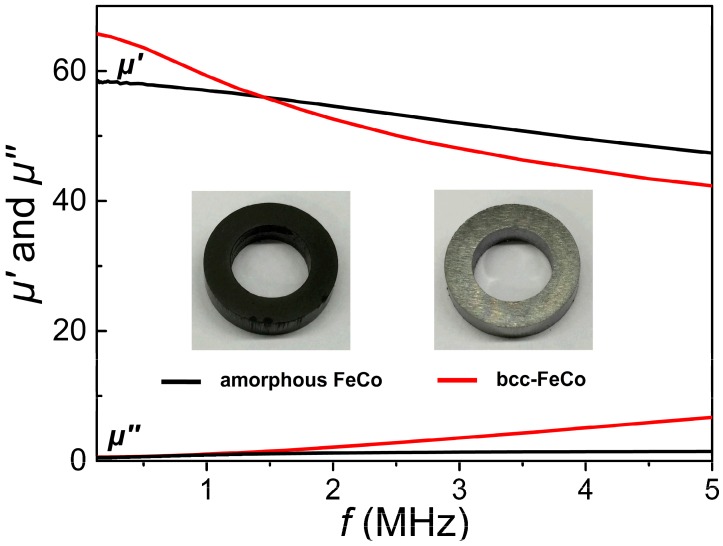
The complex permeability spectra for the two compacted samples of the amorphous FeCo prepared with 1.5 g PVP additions and high-purity Fe_65_Co_35_ particles.

For investigating soft magnetic properties of the as-synthesized amorphous FeCo product, a full-density toroidal sample (shown in the inset of Figure 8) has been produced by directly compacting the amorphous product prepared with 1.5 additions under a static pressure of approximately 1800 MPa. Another toroidal crystalline FeCo sample with the same thickness of 3 mm has also been fabricated by compacting high-purity Fe_65_Co_35_ particles with an average size of approximately 120 μm (Commercial products, purchased from Titd Metal materials Co., Ltd., Changsha, China) under the same pressure. The fully compacted amorphous FeCo sample exhibits a high density of 7.20 g/cm^3^ due to its high purity, but comparatively lower than that of the compressed micron-sized Fe_65_Co_35_ particles under the same pressure (7.62 g/cm^3^), which can be ascribed to the strong aggregation forces occurring in the compressed nanoparticles [45]. Figure 8 shows frequency-dependent complex permeability (the real part *μ*’ and imaginary part *μ*”) measured at frequencies from 40 Hz to 5 MHz for the two toroidal samples. Although the permeability and core loss of a toroidal core strongly depend on its thickness, in this work, the different influences of thickness on the permeability and core loss of the two samples can be ignored due to their same thickness. The complex permeability properties of these two toroidal cores with the same thickness can be investigated to effectively compare their closed-loop magnetic properties. From Figure 8, the amorphous FeCo product exhibits slightly lower *μ*’ values than those of crystalline FeCo sample at frequencies below 1.5 MHz, but higher *μ*’ values at frequencies from 1.5 to 5 MHz, which indicates that the amorphous sample possesses a better ac permeability. The lower *μ*’ values at low frequencies for amorphous sample can be ascribed to its relatively low *M*_s_ value, high *_i_H*_c_ value and lower density resulting from its weak surface oxidation and low surface B aggregation [2]. Furthermore, the variation of *μ*’ values with increasing frequency for amorphous FeCo product shows a gradual descending trend and very low *μ*” values at operating frequencies up to 5 MHz are also observed, indicating lower core loss in the amorphous FeCo product, which can be ascribed to its much higher electrical resistivity (approximately 1.2–2.4 × 10^−4^ Ω·cm) [46,47] than that of high-purity FeCo (1–10 × 10^−6^ Ω·cm) [48]. Nevertheless, the amorphous FeCo sample exhibits better frequency-dependent magnetic characteristics with comprehensive high *μ*’ and low *μ*” values at operating frequencies up to 5 MHz. From the above discussion, it can be concluded that these amorphous FeCo products possess high stability, good intrinsic and frequency-dependent magnetic characteristics, and show their significant potential applications in high-power magnetic devices.

## 4. Conclusions

The fully amorphous FeCo nanoalloys with low surface modification have been synthesized by a direct chemical reduction reaction of both Fe^2+^ and Co^2+^ ions with NaBH_4_ as the reductant along with PVP as the surfactant. The PVP additions have been proved to promote the formation of amorphous FeCo phase with high purity. The suitable PVP additions of 1.5 g can lead to the formation of uniform amorphous FeCo nanoparticles with a narrow size distribution and low surface oxygen content, which result in their high saturation magnetization of 196.2 A·m^2^·kg^−1^ and low coercivity of 83.3 Oe. The chemically synthesized FeCo nanoparticles exhibit high transition temperatures of 464–468 °C, which can be ascribed to their low surface B concentration. The full-density amorphous FeCo toroidal sample with a high density of 7.20 g/cm^3^ can be obtained by directly compacting the as-synthesized amorphous FeCo nanoalloys under a static pressure of 1800 MPa. Good frequency-dependent magnetic characteristics are also achieved in the fully compacted amorphous FeCo sample. The chemical synthesis for amorphous FeCo nanoalloys in this work may also provide a new insight for fabrication of metallic glasses or amorphous alloys.

## Figures and Tables

**Table 1:** The Fe/Co/O ratios measured by energy dispersive spectroscope (EDS), Fe/Co/B ratios detected by inductively coupled plasma optical emission spectroscopy (ICP-OES), average particle sizes and intrinsic magnetic properties including the values of saturation magnetization *M*_s_ and coercivity *_i_H*_c_ of amorphous FeCo nanoparticles prepared with different PVP additions. The saturation magnetization *M*_s_ value is defined as the *M* value corresponding to the maximum magnetic field of 10 KOe in Figure 7 below. The errors of the EDS, ICP-OES and vibrating sample magnetometer (VSM) measurement results are within 2%, 2% and 5%, respectively.

Samples Prepared with Different PVP Addition (g)	The Fe/Co/O Ratio (at. %) Measured by EDS	The Fe/Co/B Ratios (at. %) Detected by ICP-OES	Average Particle Sizes (nm)	*M*_s_ (A·m^2^/kg)	*_i_H*_c_ (Oe)
(a) 1 g	60.01:32.31:7.68	59.84:31.60:8.56	20	175.0	100.4
(b) 1.5 g	60.78:33.46:5.76	62.77:30.11:7.12	16	196.2	83.3
(d) 2 g	62.12:30.98:6.90	59.67:32.25:7.98	25	182.3	108.5
